# A demographic dividend of the FP2020 Initiative and the SDG reproductive health target: Case studies of India and Nigeria

**DOI:** 10.12688/gatesopenres.12803.2

**Published:** 2018-07-12

**Authors:** Qingfeng Li, Jose G. Rimon

**Affiliations:** 1 Bill & Melinda Gates Institute for Population and Reproductive Health Department of Population, Family and Reproductive Health, Johns Hopkins Bloomberg School of Public Health, Baltimore, MD, 21205, USA

**Keywords:** demographic dividend, FP2020, SDG, Nigeria, India

## Abstract

**Background:** The demographic dividend, defined as the economic growth potential resulting from favorable shifts in population age structure following rapid fertility decline, has been widely employed to advocate improving access to family planning. The current framework focuses on the long-term potential, while the short-term benefits may also help persuade policy makers to invest in family planning.

**Methods:** We estimate the short- and medium-term economic benefits from two major family planning goals: the Family Planning 2020 (FP2020)’s goal of adding 120 million modern contraceptive users by 2020; Sustainable Development Goals (SDG) 3.7 of ensuring universal access to family planning by 2030. We apply the cohort component method to World Population Prospects and National Transfer Accounts data. India and Nigeria, respectively the most populous Asian and African country under the FP2020 initiative, are used as case studies.

**Results:** Meeting the FP2020 target implies that on average, the number of children that need to be supported by every 100 working-age people would decrease by 8 persons in India and 11 persons in Nigeria in 2020; the associated reduction remains at 8 persons in India, but increases to 14 persons in Nigeria by 2030 under the SDG 3.7. In India meeting the FP2020 target would yield a saving of US$18.2 billion (PPP) in consumption expenditures for children and youth in the year 2020 alone, and that increased to US$89.7 billion by 2030. In Nigeria the consumption saved would be US$2.5 billion in 2020 and $12.9 billion by 2030.

**Conclusions:** The tremendous economic benefits from meeting the FP2020 and SDG family planning targets demonstrate the cost-effectiveness of investment in promoting access to contraceptive methods. The gap already apparent between the observed and targeted trajectories indicates tremendous missing opportunities. Accelerated progress is needed to achieve the FP2020 and SDG goals and so reap the demographic dividend.

## Introduction

Access to family planning is a critical component of reproductive rights. Family planning also provides multi-faceted benefits to women and their families. It is unique among medical interventions in its breadth of health, developmental and economic benefits, such as reducing maternal and child mortality, empowering women and girls, and enhancing environmental sustainability ^1^,^2^. The Lancet series on family planning in 2012 documented clearly the extensive gains resulting from family planning. For instance, Ahmed and colleagues estimated that in 2008 contraceptive use in 172 countries averted 272,040 maternal deaths, and that satisfying unmet need for contraceptive methods could prevent another 104,000 deaths per year ^2^. Cleland *et al*. made nearly identical estimates using a different methodology ^3^. Additionally, Canning and Schultz evaluated the economic consequences of family planning, including increases in female labor force participation and proportion of women in paid employment ^4^.

However, after reaching a global peak following the 1994 International Conference on Population and Development (ICPD) in Cairo, both financial support and political commitment for family planning have been insufficient, and they even declined in the decade prior to 2012 ^1^,^5^. On the other hand, the need for family planning increased due to population growth. With falling funding and growing population, the gap widens at both ends. Consequently, progress to increase access to contraception in developing countries has been slow. Women in sub-Saharan Africa, for example, continue having an average of more than five children ^6^. Improvement of family planning related indicators in low- and middle-income countries has lagged behind that of indicators in other development sectors, such as education, child survival and infectious disease control. Contraceptive prevalence has increased by only 0.1% points annually during the first decade of the 21 century ^7^. The reduction of maternal mortality fell short of its 2015 Millennium Development Goal (MDG) ^8^.

Two major arguments have been made to promote investments in reproductive health: human rights and economic development. The first considers reproductive health to be a fundamental human right that should be delivered for its intrinsic value ^9^. The second argues its instrumental value, particularly for stimulating economic growth and accelerating poverty reduction. A rapid reduction in fertility rate implies fewer births, and consequently a decline in dependency ratio (number of young and old dependents to working-age population). Taking advantage of the economic opportunities arising from a favorable change in the population age structure is termed “the demographic dividend.” ^10^ This concept focuses on changes in age structure, resulting from rapid fertility decline, which allow governments to shift scarce public resources from social maintenance programs such as daycare, housing, and foods to investments that better promote long-term development. A related framework that addresses the consequences of demographic change for consumption and income patterns over the life cycle, including for older populations, has brought the compound effect of early life investments into sharper relief ^11^. Implicitly, health reproduction is embedded in the fertility and mortality transitions that make possible the demographic dividend.

To improve access to contraceptive methods and to protect the rights of women and girls, a family planning summit in July 2012 launched the Family Planning 2020 (FP2020) Initiative, aiming to reach another 120 million women in the 69 poorest countries with modern contraceptives by 2020 ^12^,^13^. As of the end of 2017, about 40 countries had made political commitments to this initiative (see http://www.familyplanning2020.org/ for a full and up-to-date list).

This study’s objective is to estimate the fertility changes and associated economic benefits that would result from reaching the target of the FP2020 Initiative, using India and Nigeria as case studies. Those countries were chosen since they are respectively the most population Asian and African country under the FP2020 Initiative, and they have the required data for this study. The forecasting effort is aligned with multiple targets in the Sustainable Development Goals (SDGs) adopted by the United Nations in September 2015. For example, Target 3.7 proposes that “ *By 2030, ensure universal access to sexual and reproductive health-care service, including for family planning.*” ^14^ Another target, under gender goal, states that “ *Ensure universal access to sexual and reproductive health and reproductive rights*.” ^14^

## Methods

To estimate the economic benefit for India and Nigeria of meeting unmet need for contraception, we compare total consumption for selected years from 2015 through 2030 under two difference scenarios of population growth and chances in age structure—one based on the projection of current contraceptive use patterns and the other based on gradually meeting all unmet need for contraception by 2030.

The contraceptive prevalence data come from World Contraceptive Use (WCU) 2015, assembled by the United Nations Development Programme (UNDP). The dataset covers modern contraceptive prevalence rate among married or in-union women aged 15-49 years (married MCPR). Fertility data were extracted from World Population Prospects (WPP) 2015, also produced by UNDP. Data on consumption are from the National Transfer Accounts (NTA) project ^15^.

In order to encourage country ownership and accountability, the FP2020 Initiative set an overall target without setting country-specific goals. To make the predictions for India and Nigeria, we assumed that all unmet need in 2012 is gradually satisfied by modern contraceptive methods by 2030. The projection is divided into two phases: 2012–2020 and 2020–2030. The first phase is based on an assumption that 64% of unmet need for family planning with modern methods is satisfied. This percentage would amount to 120 million additional married users of modern contraceptives in the 69 poorest countries by 2020—the FP2020 goal. Data on unmet need are available only for married women; therefore, we are not able to replicate exactly the FP2020 goal covering both married and sexually active unmarried women. As a result, our estimate is more conservative than the actual FP2020 target. We assume that the remaining 36% of unmet need is satisfied in the second phase, 2020–2030. Using the numbers of married women as projected in the UN database, we calculate the number of additional modern contraceptive users and the percentage of the demand for family planning satisfied by modern methods.

Our analytical approach has three main steps. First, we estimated the impact of increased modern contraceptive prevalence rate (mCPR) on the population age structure. The fertility change is calculated by the general fertility rate (GFR), defined as the yearly number of births per 1000 women of reproductive age. We chose GFR instead of total fertility rate (TFR), which is the number of children per woman if she was to experience the assumed aged-specific fertility rates, because GFR accounts for the age distribution of women. This is in line with the definition of the model covariate mCPR, which is the weighted average of age-specific contraceptive use rates with the age distribution of women as the weight. A quadratic relationship between GFR and mCPR was estimated ^16^:

$$GFR_{t}=\beta_{0}+\beta_{1}*mCPR_{t}+\beta_{2}*mCPR_{t}^{2}+\in_{t}\;(1)$$

where *GFR _t_* denotes the GFR at time *t*; *MCPR _t_* denotes the mCPR at time *t*; *β*_0_, *β*_1_, *β*_2_ denotes coefficients; and *∈ _t_* denotes the random error term. Then with the estimated coefficients we predicted the GFR using the simulated mCPR under the FP2020 Initiative and SDG target.

In the second step we applied the estimated GFR to the population age structures from 2012 to 2020, employing the cohort component method (CCM). This is the demographic projection method used to generate World Population Prospects (WPP). Based on a transition matrix, population by age is projected from one period to the next. Following WPP 2015, our projections were made for five-year intervals up to age 90+. The basic equation for the CCM projection is:

$$P_{t+1}=M_{t,t+5}*P_{t}\;(2)$$

where *P* *_t_* is a column vector with elements denoting the age group-specific population at calendar time *t*; *P*_*t*+1_ is the population vector for time *t* + 5 . *M*_*t*, *t*+5_ is a transition matrix constructed from age-specific fertility and mortality rates. Its elements, denoting fertility and survivorship at respective ages, are used to determine the births and deaths for each age group in each year. The age-specific survival probability was assumed to be constant over time. While this is not realistic, it serves our purpose of estimating the effect of fertility change while controlling for other determinants of population growth.

In the third and last step, we applied the age-specific consumption data available from the National Transfer Accounts (NTA) project. The difference in total consumption between the UN scenario and the FP2020 scenario is considered to be an economic benefit of meeting the FP2020 goal of increased mCPR and the reduction in fertility that results.

The available NTA estimates for India and Nigeria are both for the year 2004, measured in US dollars (converted from Indian rupees and Nigerian naira using purchasing power parity (PPP)). As the economies of both countries are projected to continue growing, consumption per capita will increase in the projection period. In Nigeria the annual growth rate of real GDP per capita was 3.62% in the five-year period 2000–2005 and 4.07% for 2005–2010 ^17^. We apply a conservative estimate of 3% annual increase to consumption per capita. In India the annual growth rate of real GDP per capita was 5.41% for 2000–2005 and 7.11% for 2005–2010. We use a conservative estimate of a 5% annual increase to adjust consumption per capita. We further assume that this rate of increase in consumption is constant across all ages. Future saving is discounted to its present value to facilitate the cost-benefit analysis of the investment. An annual 3% discount rate was used to convert future savings to present discounted value (PDV), presented in Table 1. A comparison of investment in RH with the PDV of the future economic benefits informs policy-makers whether the allocation of resources is wise.

**Table 1.** Estimated present discounted value (PDV) of total consumption and consumption savings through gradually eliminating unmet need by 2030 (in billions of US$, discounted to 2014).

Country	Year	Without FP2020 and SDG influences		Under FP2020 and SDG			
		Youth dependency ratio	Total consumption	Youth dependency ratio	Total consumption	Averted consumption	Averted consumption (%)
India	2015	43.4	2,520.0	38.8	2,518.2	1.8	0.07
	2020	43.2	2,962.9	34.9	2,944.7	18.2	0.62
	2025	40.7	3,451.6	36.4	3,403.1	48.5	1.40
	2030	38.8	3,986.4	31.3	3,896.7	89.7	2.25
Nigeria	2015	84.8	225.4	81.7	225.2	0.3	0.11
	2020	84.3	258.2	73.8	255.6	2.5	0.98
	2025	84.2	294.4	76.8	287.6	6.9	2.34
	2030	80.4	334.2	66.0	321.3	12.9	3.86

FP2020: Family Planning 2020; SDG: Sustainable Development Goals

All statistical analyses were done with Stata (version 15).

## Results

**Figure 1.**
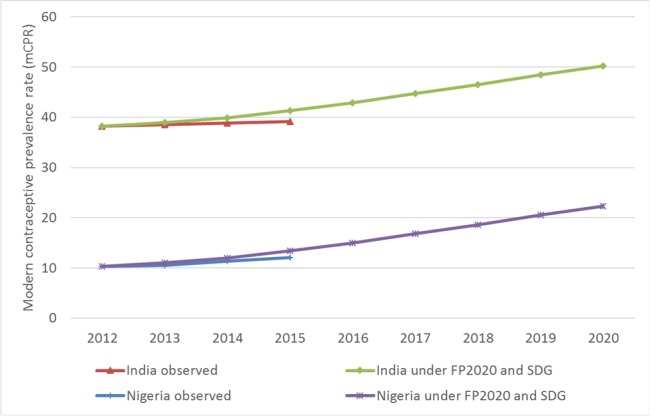
The trajectory of modern contraceptive use rates in India and Nigeria: observed and targeted.

**Figure 2.**
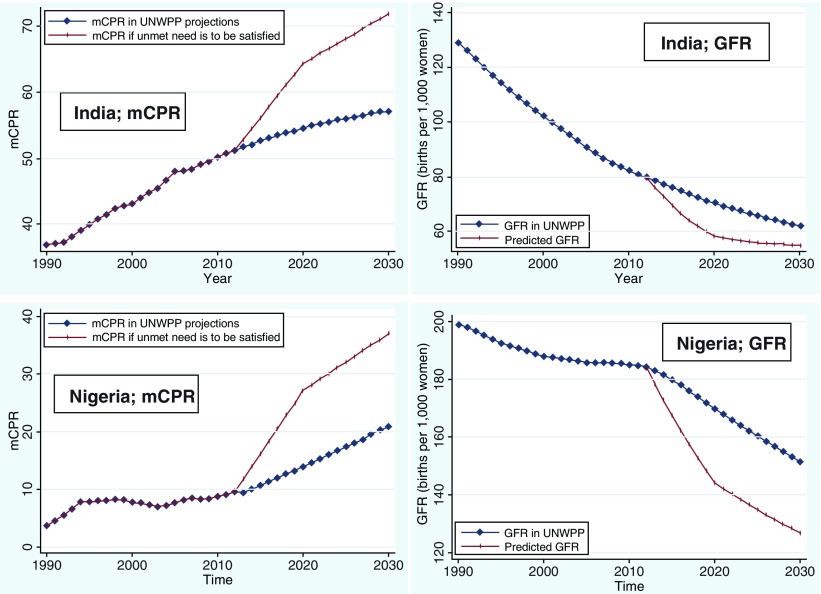
Trends of modern contraceptive prevalence rate (mCPR) and general fertility rate (GFR), 1990–2030, in India and Nigeria in UN World Population Prospects, 2015 and scenario of satisfying unmet need by 2030.

The observed mCPR to date is below the FP2020 targeted trajectory in both countries ( Figure 1). The difference in mCPR implies missed opportunities in shifting the population age structure and reaping a demographic dividend. Figure 2 illustrates the trends of mCPR and GFR in India under the WPP projection and our assumed scenario of achieving the FP2020 target by 2020 and then meeting the SDG 3.7 goal of eliminating unmet need by 2030. The wide gap between the two curves calls for urgent effort and investment to accelerate progress in mCPR. The situation in Nigeria looks similar ( Figure 2).

Table 1 presents the youth dependency ratio (YDR) under the two scenarios: the WPP forecast and the FP2020 Initiative. YDR is defined as the ratio of the number of children ages 0–14 years to the working population ages 15–64 years. The UN predicts an YDR of 43.2 in 2020 for India. Under the FP2020 scenario, the YDR would be reduced to 34.9. In other words, on average, the number of children that need to be supported by every 100 working-age people would decrease by 8 persons by following the FP2020 scenario. In Nigeria the YDR would drop by 10 in 2020, from the 84.3 predicted by the UN to 73.8 under FP2020. The resource savings at both household and national levels could be used to improve the quality of human capital and foster productive investments. This would translate into improved living conditions in both the short and long terms.

The economic benefits of satisfying unmet need are substantial in both countries. As Table 1 shows, India would save US$18.2 billion (PPP) in consumption expenditures in 2020 if the FP2020 goal were to be achieved. In Nigeria the consumption expenditures saved would be US$2.5 billion (PPP) in 2020. These estimates have obvious implications for the cost-benefit analysis of FP2020 and are consistent with those reported by previous studies ^18^,^20^.

Clearly, the consumption expenditures saved is only one of many economic benefits of FP2020. Numerous studies have provided evidence for other social benefits of increased use of contraceptives ^21^,^22^. Our estimates show that, even considering only the consumption expenditure savings, investment in the goals outlined by FP2020 will be a wise use of resources in both countries.

## Discussion

To our knowledge, this is the first economic evaluation of the FP2020 and SDG family planning goals. Those goals are quite ambitious, considering the slow progress towards increasing mCPR in the decade prior to FP2020. Greater financial investment and a stronger political will are crucial to the success of the Initiative. By demonstrating one of the many economic benefits of increased mCPR and consequently reduced fertility, this paper hopes to encourage greater investment in family planning programs and to underscore the need to foster the stronger political will necessary to ensure achievements in this field. The tremendous savings on consumption expenditure could be invested in many other areas, such as making better education available and affordable to more people, particularly girls, and building more infrastructure needed to stimulate further economic development. At the same time, greater access to contraception would save more women’s lives, improve the health of many more women and their children, promote economic empowerment particularly for women, and enable people to exercise their fundamental human right to determine for themselves the number and spacing of their children.

## Data availability

All data used in the study are freely available online (no registration needed). Below are links to access the datasets:

- World Contraceptive Use 2015: http://www.un.org/en/development/desa/population/publications/dataset/contraception/wcu2015.shtml
- World Population Prospects 2015: https://esa.un.org/unpd/wpp/
- National Transfer Account: http://www.ntaccounts.org
