# Supplementary material for: A demographic dividend of the FP2020 Initiative and the SDG reproductive health target: Case studies of India and Nigeria
Source: Gates Open Res. 2018 Jul 12;2:11. Originally published 2018 Feb 22. [Version 2] doi: 10.12688/gatesopenres.12803.2 (PMC5883068; doi:10.12688/gatesopenres.12803.2)
Supplement: Peer review history

# Peer review: Referee response for version 1

**DOI:** 10.21956/gatesopenres.13867.r26331

**Published:** 2018-04-16

**Authors:** Akanni Akinyemi

**Affiliations:** 1 Department of Demography and Social Statistics, Obafemi Awolowo University, Ile Ife, Nigeria

The article reinforces the importance on investment in family planning for economic growth as part of ongoing discourse on demographic dividend. The choice of the two countries for the model is appropriate and well justified. However, beyond the call for investment in education education and family planning, there is the need for economic empowerment particularly for women in order for both countries to reap her demographic dividend.

I have read this submission. I believe that I have an appropriate level of expertise to confirm that it is of an acceptable scientific standard.

# Response

**Published:** 2018-07-06

**Authors:** Qingfeng Li

Thanks for taking time to review our manuscript. We agree with with reviewer that the realization of the projected economic benefits is not automatic, but requires policy interventions, such as empowering women. We have revised the manuscript to incorporate this suggestion.

# Peer review: Referee response for version 1

**DOI:** 10.21956/gatesopenres.13867.r26332

**Published:** 2018-04-03

**Authors:** Baochang Gu

**Affiliations:** 1 Center for Population and Development Studies, Renmin University of China, Beijing, China

The short essay carried an exercise to demonstrate how the increase in contraceptive use by the goals set up by the FP2020 initiative and SDG2030 target, may bring down fertility and create a shift in population age structure favourable to economic growth.

 It is appropriate to pick up India and Nigeria for case studies given that they are “the most populous Asia and African country under the FP2020 initiative”.  But meanwhile, it should be noted that between the two countries comparatively India’s fertility is much lower and contraceptive use is much higher while the potential in population growth is much greater in Nigeria.  The differences at the onset may affect their prospect when embarking on toward the FP2020 initiative and SDG2030 target.

 To display the changes in age structure the authors may consider to constructing the population pyramids for the countries from present to 2020 and to 2030, which may make it more intuitive to policy makers as well as the general public.

 It will be helpful to say a few words what is GFR and why it is chosen as fertility indicator in the exercise.

I have read this submission. I believe that I have an appropriate level of expertise to confirm that it is of an acceptable scientific standard.

# Response

**Published:** 2018-07-06

**Authors:** Qingfeng Li

Thanks for the suggestions. We have added the definition for GFR and explained why we chose GFR over TFR as a fertility measure. 

 Pyramid is a great visualization tool particularly for policy makers, but unfortunately we are not able to make pyramids in this study because our estimation is not disaggregated by sex.

# Peer review: Referee response for version 1

**DOI:** 10.21956/gatesopenres.13867.r26302

**Published:** 2018-04-03

**Authors:** Kaushalendra Kumar Singh

**Affiliations:** 1 Department of Statistics, Institute of Science, Banaras Hindu University, Varanasi, India

During the recent years, the issue of demographic dividend has received wide attention from economists, demographers, policymakers. However, most of the discussion has centered on harvesting the dividend and the timing of dividend, in particular, the timing of opening and closing the window of opportunity. This paper addresses an important aspect of dividend, namely, the tremendous economic benefits from meeting the FP2020 and SDG family planning targets demonstrate the cost-effectiveness of investment in promoting access to the role of modern family planning in the dividend.

 The paper is a valuable addition to the literature on demographic dividend and I recommend it for acceptance after incorporating/clarifying following points.

 A few points for the consideration of the author(s) are given below.

  

1. India is country where traditional method of contraceptive is also popularly used in the society, which should be taken into account.
2. TFR may be used to calculate fertility change in the place of GFR.
3. In equation 1, I think something is missing. Correct it.

I have read this submission. I believe that I have an appropriate level of expertise to confirm that it is of an acceptable scientific standard.

# Response

**Published:** 2018-07-06

**Authors:** Qingfeng Li

Thanks for the suggestions. 

 Traditional contraceptive methods are not accounted for in this study because both FP2020 and SDG focus on modern methods. 

 As noted in our response to reviewer #2, we have included a justification of using GFR instead of TFR in this study.

 Equation 1 seems correct to us , but we have added notation definitions below the equation. Hopefully that clarifies the model specification.
